# A pair of new BAC and BIBAC vectors that facilitate BAC/BIBAC library construction and intact large genomic DNA insert exchange

**DOI:** 10.1186/1746-4811-7-33

**Published:** 2011-10-11

**Authors:** Xue Shi, Haiyang Zeng, Yadong Xue, Meizhong Luo

**Affiliations:** 1 National Key Laboratory of Crop Genetic Improvement, Huazhong Agricultural University, Wuhan, 430070, China

**Keywords:** BAC, BIBAC, positional cloning, *Agrobacterium*, maize

## Abstract

**Background:**

Large-insert BAC and BIBAC libraries are important tools for structural and functional genomics studies of eukaryotic genomes. To facilitate the construction of BAC and BIBAC libraries and the transfer of complete large BAC inserts into BIBAC vectors, which is desired in positional cloning, we developed a pair of new BAC and BIBAC vectors.

**Results:**

The new BAC vector pIndigoBAC536-S and the new BIBAC vector BIBAC-S have the following features: 1) both contain two 18-bp non-palindromic I-*Sce*I sites in an inverted orientation at positions that flank an identical DNA fragment containing the *lac*Z selection marker and the cloning site. Large DNA inserts can be excised from the vectors as single fragments by cutting with I-*Sce*I, allowing the inserts to be easily sized. More importantly, because the two vectors contain different antibiotic resistance genes for transformant selection and produce the same non-complementary 3' protruding ATAA ends by I-*Sce*I that suppress self- and inter-ligations, the exchange of intact large genomic DNA inserts between the BAC and BIBAC vectors is straightforward; 2) both were constructed as high-copy composite vectors. Reliable linearized and dephosphorylated original low-copy pIndigoBAC536-S and BIBAC-S vectors that are ready for library construction can be prepared from the high-copy composite vectors pHZAUBAC1 and pHZAUBIBAC1, respectively, without the need for additional preparation steps or special reagents, thus simplifying the construction of BAC and BIBAC libraries. BIBAC clones constructed with the new BIBAC-S vector are stable in both *E. coli *and *Agrobacterium*. The vectors can be accessed through our website http://GResource.hzau.edu.cn.

**Conclusions:**

The two new vectors and their respective high-copy composite vectors can largely facilitate the construction and characterization of BAC and BIBAC libraries. The transfer of complete large genomic DNA inserts from one vector to the other is made straightforward.

## Background

High-quality, deep-coverage, large-insert genomic libraries are important tools for structural and functional genomics studies of eukaryotic genomes. The BAC (bacterial artificial chromosome) cloning system [1] has been used to construct the most of such genomic libraries for different organisms that include important crops and model plants, such as rice [2,3], maize [4], wheat [5], soybean [6], barley [7] and Arabidopsis [8]. BAC libraries have been used for physical mapping [3,6,9], BAC to BAC genome sequencing [8,10,11], positional cloning [12-16], comparative genomics [17,18], and genome assemblies of whole genome shotgun sequences [19] and next-generation sequences [20]. To facilitate the positional cloning of plant genes, BIBAC (binary BAC) and TAC (transformation-competent artificial chromosome) vectors were developed to clone and transfer large-insert DNA fragments into plants via *Agrobacterium*-mediated transformation [21,22]. The BIBAC vector contains the BAC vector backbone that uses the F-plasmid origin for replication in *E. coli*. The TAC vector uses the *E. coli *bacteriophage P1 origin for replication in *E. coli*. Both vectors use the single-copy Ri origin from *Agrobacterium rhizogenes *for replication in *Agrobacterium*. Several BIBAC and TAC libraries have been constructed such as those for rice [23-26], chickpea [27], tomato [25,28], sunflower [29], Arabidopsis [22,30], wheat [31] and banana [32]. However, although in addition to positional cloning BIBAC and TAC libraries could be also used for general purposes such as physical mapping and genome sequencing that BAC libraries are used for, BAC libraries were more popularly used than BIBAC and TAC libraries because the BIBAC and TAC vectors (~23 kb) have a larger size than the BAC vector (~7.5 kb), which increases difficulties for large DNA fragment library construction [25] and costs for repeated shotgun sequencing of the vector sequences. For the positional cloning of genes, re-cloning of BAC inserts into BIBAC or TAC vectors is often required for gene function complementation [13-15,33].

The vectors and technologies associated with the construction of large genomic DNA fragment libraries are constantly improving. Before the BAC cloning system [1] was established, the YAC (Yeast artificial chromosome) cloning system [34] was used. However, the YAC cloning system had several disadvantages, such as high levels of chimerism and difficulty of handling. The BAC cloning system overcomes many of these disadvantages and advanced methods for the construction of BAC libraries have been developed [35-38]. The BAC vector uses the F-plasmid origin of replication in *E. coli *and has a low copy number (1-2 copies per cell). While a low-copy replication is considered important for the stable maintenance of large DNA fragments in *E. coli *[1], vector preparation was difficult due to the low yield of DNA. To facilitate the preparation of vector DNA, BAC vectors have been modified using different strategies. Frengen et al. [39] constructed a high-copy BAC vector (pBACe3.6) by inserting the high-copy pUC vector into the cloning site of the BAC vector (a pBAC108L-derivative). The original BAC vector can be recovered from large-scale DNA preparations of pBACe3.6 and used for the construction of BAC libraries. However, because the pBACe3.6 colonies cannot be distinguished from the recombinant BAC clones by selection, care must be taken to avoid the contamination of the BAC library with the high-copy pBACe3.6 vector [39]. Wild et al. [40] engineered a conditional amplification system for the BAC vector. These researchers inserted a high-copy replication origin, *oriV*, into the BAC vector and inserted the gene coding for TrfA replication protein under the control of the inducible araC-*Para*BAD promoter/regulator system into the host genome. Replication at *oriV *is dependent on the expression of TrfA. Following the induction of TrfA protein expression by L-arabinose, the BAC vector or the BAC clones that were constructed using this vector replicated at a high copy number. The vector is approximately 500 bp larger than the original BAC vector and only functions within the engineered host cells. Previously, we constructed a high-copy composite BAC vector, pCUGIBAC1, which contains the low-copy BAC vector pIndigoBAC536 ligated to the high-copy vector pGEM-4Z [37,41]. As a vector, most important is that it can be distinguished from the recombinant clones by selection. Two special features assured the composite vector pCUGIBAC1 of a reliable vector. First, the pIndigoBAC536 and the high-copy pGEM-4Z vectors each contain a *lacZ *gene of the same origin. Therefore, of the two ligation products between the two vectors (head-head and head-tail), one ligation product (head-tail) can reconstitute two *lacZ *gene copies. Second, of the two ligation products, only one can replicate in *E. coli*. The pCUGIBAC1 took the advantages of both features and so the grown colonies were all blue on X-gal-containing selection medium [37]. The pCUGIBAC1 replicated at a high-copy number and largely facilitated BAC vector preparations. The pCUGIBAC1 DNA can be digested with *Hin*dIII, *Bam*HI or *Eco*RI to produce linearized forms of the original pIndigoBAC536 and pGEM-4Z vectors [41]. Therefore, linearized and dephosphorylated form of the original BAC vector pIndigoBAC536 that retains all of its original features can be prepared from the high-copy composite vector pCUGIBAC1 by restriction digestion without the need for additional preparation steps or special reagents. Because any self- or inter-ligation products of the pIndigoBAC536 and the pGEM-4Z fragments regenerate the pIndigoBAC536, pGEM-4Z or pCUGIBAC1 plasmids, whose transformants are all blue and distinguishable from the recombinant BAC clones (white) on X-gal-containing selection medium, trace amounts of the pCUGIBAC1 and/or the pGEM-4Z fragments trapped during the preparation of the pIndigoBAC536 vector will not cause contamination of the BAC libraries [37]. The pIndigoBAC536 vector prepared from the pCUGIBAC1 was used to construct many BAC libraries such as those for 12 *Oryza *species [17], nurse shark [42], zebra finch [43] and 19 *Drosophila *species [44].

However, despite these developments, vectors that are currently available for the construction of large-insert genomic libraries still have limitations. It is difficult to obtain enough BIBAC plasmid DNA for vector preparation and there have been no attempts to modify the BIBAC vector to increase its copy number. Commonly used BAC and BIBAC vectors contain two *Not*I restriction sites that flank the multiple cloning sites for insert sizing and releasing. *Not*I is a rare-cut restriction enzyme that recognizes the 8-bp sequence GCGGCCGC. *Not*I digestion of BAC/BIBAC clones of libraries originating from organisms with low GC content results in a few large insert bands per clone when viewed on a CHEF gel. However, for BAC/BIBAC clones that originate from organisms with high GC content, such as monocotyledonous plants, *Not*I digestion produces many small DNA fragments per clone and therefore, insert sizing is difficult and transfer of intact inserts from one vector to another is almost impossible [32,33,45]. Insert sizing is an important step that determines the quality of large DNA fragment libraries [41] and for comparative genomics [46]. Genome expansions and contractions can be estimated by comparing the actual insert sizes of the BAC contigs to the corresponding regions of a reference sequence [46]. Because genetic mapping usually cannot locate a gene in a narrow region [22], without a method to re-clone large BAC inserts into the BIBAC vector in one piece, the large BAC inserts must be fragmented and sub-cloned into a binary vector, and the individual sub-clones should be used to transform plants [32,33]. This process contributes to an increase in the labor, costs and complexity of the procedure. The TAC vector series [22,23,25] contains two 18-bp recognition sites for the homing endonuclease I-*Sce*I that flank the cloning site and the plant selection marker. The I-*Sce*I sites can be used for insert sizing of the TAC libraries and to examine the integrity of the transferred inserts in the transgenic plants by digesting DNA from putative transgenic plants with I-*Sce*I and hybridizing with a probe for the plant selection marker. The TAC vector series also contains a P1 lytic replicon, and the copy number can be amplified by releasing the suppresser of the P1 lytic replicon with IPTG (Isopropyl-β-D-thiogalactoside) [22]. However, the I-*Sce*I sites cannot be used to directly clone DNA sequences or to re-clone the BAC inserts for *Agrobacterium*-mediated transformation because the plant selection marker is not located on the vector backbone.

In this study, we constructed a pair of BAC and BIBAC vectors that overcome the above limitations.

## Results

### Construction of new BAC and BIBAC vectors

**Figure 1:**
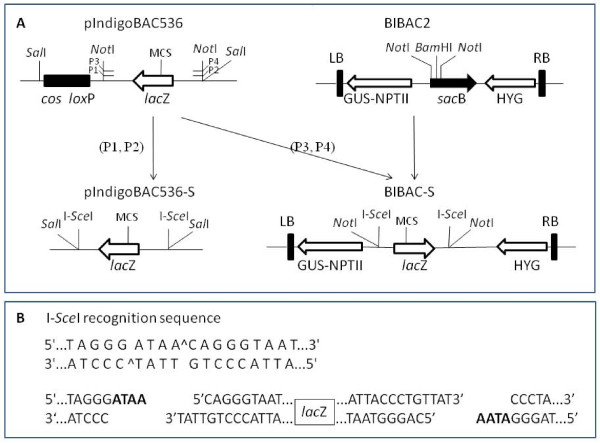
**Construction of the new BAC vector pIndigoBAC536-S and the new BIBAC vector BIBAC-S (diagram is not to scale)**. A. Schematic outline for the construction of the two new vectors. The *Not*I fragment of pIndigoBAC536 was amplified by PCR using P1/P2 primers that contained *Sal*I and I-*Sce*I recognition sites at the 5' ends and P3/P4 primers that contained *Not*I and I-*Sce*I recognition sites at the 5' ends. The PCR products were ligated to the *Sal*I- digested backbone of pIndigoBAC536 and the *Not*I-digested backbone of BIBAC2, resulting in the plasmids pIndigoBAC536-S and BIBAC-S, respectively. MCS: multiple cloning sites. *Hin*dIII, *Bam*HI and *Eco*RI are three unique sites in pIndigoBAC536 and pIndigoBAC536-S used for BAC library construction. *Bam*HI is the unique site in BIBAC2 and BIBAC-S used for BIBAC library construction. The MCS also contains a *Sal*I site. B. The sequence, orientation and locations of the I-*Sce*I sites in the pIndigoBAC536-S and BIBAC-S vectors. The two I-*Sce*I sites are located in an inverted orientation at positions that flank an identical DNA fragment containing the *lac*Z selection marker and the cloning site. After digestion by I-*Sce*I, both vectors produce the non-complementary ATAA ends (in bold).

**Figure 2:**
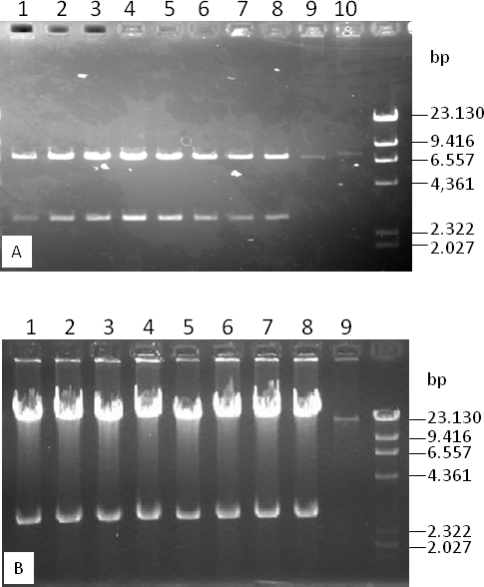
**DNA analyses of randomly picked colonies showing copy number increases of the composite vectors pHZAUBAC1 and pHZAUBIBAC1**. A. DNA analysis of randomly picked colonies transformed with pIndigoBac536 (lane 10), pIndigoBac536-S (lane 9) and the ligation product of pIndigoBac536-S and pGEM-4Z at the *Hin*dIII cloning site (pHZAUBAC1, lanes 1-8). DNA samples were prepared from 2 mL overnight cultures and were resuspended in 50 μL TE. Aliquots of 5 μL were digested with *Hin*dIII and were loaded onto a 1% agarose gel. pIndigoBAC536-S is 470 bp smaller than pIndigoBAC536. B. DNA analysis of randomly picked colonies transformed with BIBAC-S (lane 9) and the ligation product of BIBAC-S and pGEM-4Z at the *Bam*HI cloning site (pHZAUBIBAC1, lanes 1-8). DNA samples were prepared from 2 mL overnight cultures and were resuspended in 50 μL of TE. Aliquots of 5 μL were digested with *Bam*HI and were loaded onto a 1% agarose gel. The marker is lambda/*Hin*dIII.

To facilitate the release and recovery of complete inserts from BAC clones, we constructed a new BAC vector by replacing the two 8-bp *Not*I restriction sites in the pIndigoBAC536 with the 18-bp homing endonuclease I*-Sce*I sites. The *Not*I fragment of pIndigoBAC536 was amplified by PCR using the primers P1 and P2 that contain *Sal*I and I*-Sce*I sites at the 5' ends. This PCR product was ligated to the *Sal*I backbone fragment (6,384 bp) of pIndigoBAC536, resulting in pIndigoBAC536-S (Figure 1). Ligation of the *Sal*I PCR fragment with the *Sal*I backbone fragment of pIndigoBAC536 produces two possible products with opposite relative orientations. The pIndigoBAC536-S is from the ligation product that contains *lac*Z and the vector backbone in the same orientation as the original pIndigoBAC635. This orientation is critical to ensure that the composite vector containing pIndigoBAC536-S and pGEM-4Z (see below) contains two reconstituted *lac*Z genes for selection [37]. As shown in Figure 1A, the lambda *cosN *and P1 *loxP *sites of pIndigoBAC536 that are not required for BAC library construction and application were removed in pIndigoBAC536-S. The final size of pIndigoBAC536-S is 7,037 bp, which is 470 bp smaller than pIndigoBAC536 (7,507 bp). As expected, the pIndigoBAC536-S plasmid can be linearized with *Hin*dIII, *Bam*HI, or *Eco*RI, can be digested into two fragments (the vector backbone and *lac*Z-containing fragments) with I-*Sce*I, and its transformants are dark blue on chloramphenicol- and X-gal-containing selection medium (data not shown). To make the low-copy pIndigoBAC536-S a high-copy composite vector, pIndigoBAC536-S was ligated to the high-copy vector pGEM-4Z at the *Hin*dIII site following a previously described protocol [37]. Ligation of pIndigoBAC536-S with pGEM-4Z could also result in two possible ligation products that have opposite relative orientations (head-head and head-tail). However, only the ligation product (head-tail) that reconstitute two *lac*Z genes can replicate in *E. coli *and all the grown colonies on the antibiotic- (12.5 μg/mL of chloramphenicol and 50 μg/mL ampicillin) and X-gal-containing selection medium are dark blue. DNA analysis of sampling colonies demonstrated that the copy number of pIndigoBAC536-S was increased by the presence of pGEM-4Z, and the intact pIndigoBAC536-S vector was released by restriction digestion with *Hin*dIII (Figure 2), *Bam*HI, or *Eco*RI (not shown). The new composite BAC vector was named pHZAUBAC1.

**Figure 3:**
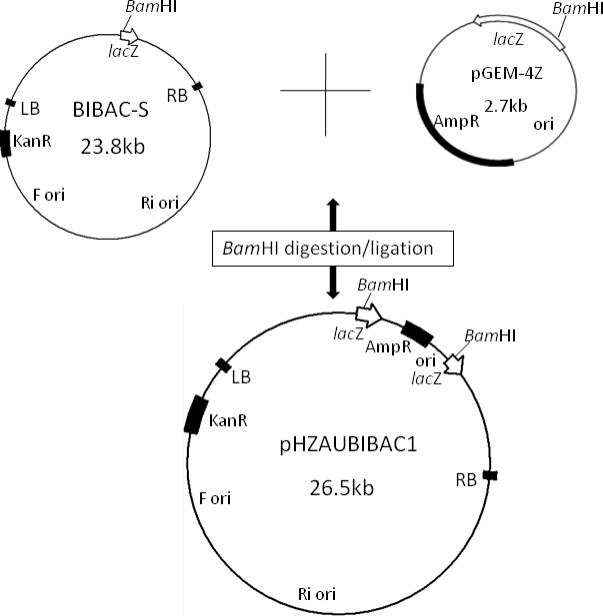
**Maps of BIBAC-S and pHZAUBIBAC1**. pGEM-4Z was purchased from Promega (Madison, Wis.).

To facilitate BIBAC library construction and insert exchange between BAC and BIBAC vectors, we also constructed a new BIBAC vector using approaches similar to those described above. The *Not*I fragment of pIndigoBAC536 was amplified by PCR using the primers P3 and P4 that contain *Not*I and I*-Sce*I recognition sites at their 5' ends and was ligated to the *Not*I backbone fragment of BIBAC2 [47] (obtained from the Cornell Center for Technology Enterprise & Commercialization), resulting in BIBAC-S (Figure 1). From the two ligation products with opposite relative orientations of *lac*Z to the vector backbone that were generated, the ligation product with the orientation of *lac*Z to the vector backbone as shown in Figure 1 was chosen for BIBAC-S. BIBAC-S is approximately 350 bp larger than BIBAC2. As in BIBAC2, the *Bam*HI restriction site is also the unique cloning site in BIBAC-S. The BIBAC-S plasmid can be linearized with *Bam*HI, can be digested into two fragments (the vector backbone and *lac*Z-containing fragments) with I-*Sce*I or *Not*I, and its *E. coli *transformants are dark blue when grown on kanamycin- and X-gal-containing selection medium. The low-copy BIBAC-S was ligated to the high-copy pGEM-4Z vector at the *Bam*HI site. As expected, all transformants of the composite BIBAC vector, named as pHZAUBIBAC1, were from the ligation product (head-tail) in which two *lac*Z genes were reconstituted and were dark blue in color when grown on antibiotic- (20 μg/mL of kanamycin and 50 μg/mL ampicillin) and X-gal-containing selection medium because the other ligation product (head-head) could not replicate in *E. coli*. DNA analysis of sampling colonies showed that the copy number of the BIBAC-S was increased by the presence of pGEM-4Z, and the original BIBAC-S was released from the composite BIBAC vector pHZAUBIBAC1 by restriction digestion with *Bam*HI (Figure 2). The map of pHZAUBIBAC1 is shown in Figure 3.

### Utility demonstration of the new BAC and BIBAC vectors

**Figure 4:**
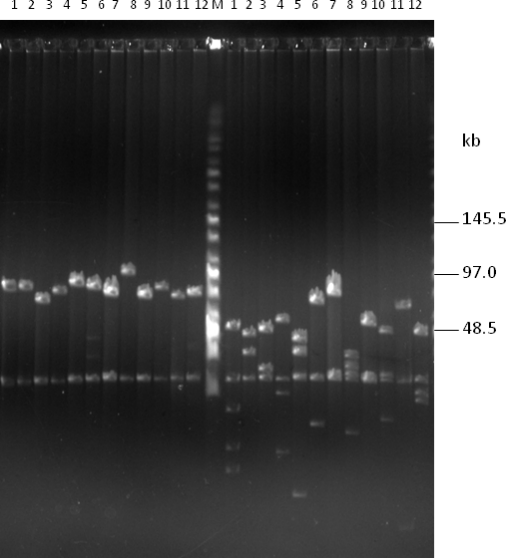
**DNA analysis of randomly selected clones from the maize B73 BIBAC library, which was constructed using the BIBAC-S vector, by pulsed-field gel electrophoresis**. DNA samples were prepared from 12 randomly selected BIBAC clones. The same set of DNA samples was digested with I-*Sce*I (left lanes 1-12) and *Not*I (right lanes 1-12) and was separated on a 1% agarose CHEF gel with a ramp pulse time of 5-15 s at 6 V/cm at 14°C in 0.5 × TBE buffer for 16 h. The molecular weight marker is Midrange I (New England Biolabs). The common 23.2-kb band is the vector BIBAC-S.

The merit of high-copy composite vector in the construction of BAC libraries has been previously described [37]. Figure 2 showed that the copy number of the low-copy pIndigoBAC536-S and BIBAC-S vectors increased 50- to 100-fold in the high-copy composite vectors. The results indicate that 200 ml of a high-copy composite vector culture can produce the same amount of vector DNA as 10-20 L of a low-copy vector culture, a minimum amount of vector DNA that is needed for BAC or BIBAC library construction. The cloning sites used in the construction of BAC (*Bam*HI, *Hin*dIII and *Eco*RI) and BIBAC (*Bam*HI) libraries can be used to recover the BAC vector pIndigoBAC536-S and the BIBAC vector BIBAC-S from the composite vectors pHZAUBAC1 and pHZAUBIBAC1, respectively. Therefore, linearized and dephosphorylated forms of the original low-copy pIndigoBAC536-S and BIBAC-S vectors, which are ready for library construction, can be prepared from their respective high-copy composite vectors without the need for additional preparation steps or special reagents, thereby simplifying the construction of BAC and BIBAC libraries. Many BAC libraries, including three maize BAC libraries, a *parviglumis *BAC library and a *tripsacum *BAC library, have been constructed using the pIndigoBAC536-S vector that was prepared from pHZAUBAC1, and a maize B73 BIBAC and a sorghum BIBAC library have been constructed using the BIBAC-S vector prepared from pHZAUBIBAC1. All of these BAC and BIBAC libraries are of high quality (unpublished data; see the resource list on our website http://GResource.hzau.edu.cn). The I-*Sce*I sites largely facilitated the characterization of the libraries. The digestion of samples of BAC and BIBAC clones with I-*Sce*I released only one insert band from each clone. Figure 4 shows the results of the digestion of 12 randomly selected maize B73 BIBAC clones with I-*Sce*I or *Not*I. In most cases, *Not*I digestion resulted in many insert fragment bands per clone due to the presence of internal *Not*I sites in the cloned genomic DNA.

**Figure 5:**
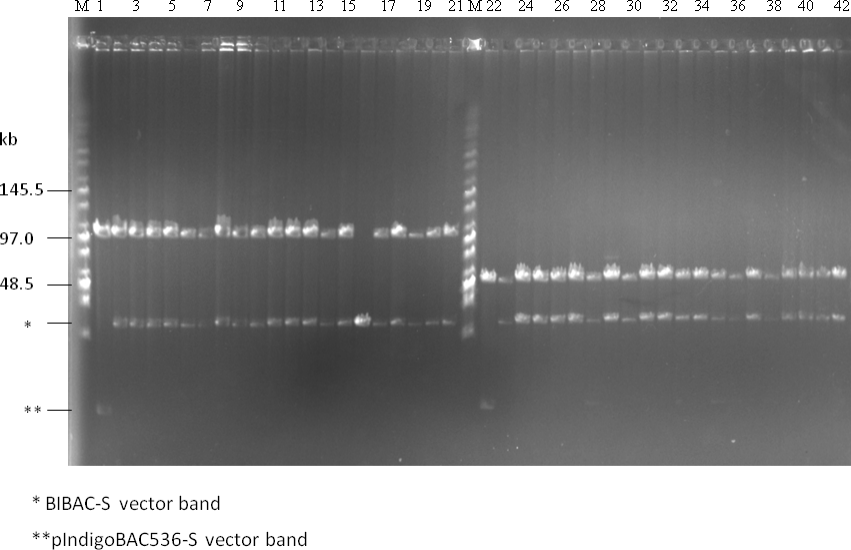
**Transfer of intact BAC inserts into the BIBAC-S vector using I-*Sce*I**. Two BAC clones with inserts of 100 kb (lane 1) and 50 kb (lane 22) were randomly selected from a maize Mo17 BAC library that was constructed using the pIndigoBAC536-S vector. DNA samples were digested with I-*Sce*I and were ligated to the BIBAC-S vector prepared with I-*Sce*I from the composite BIBAC vector pHZAUBIBAC1. The ligation products were used to transform *E. coli *DH10B competent cells. DNA samples from twenty randomly selected colonies for each ligation were analyzed with I-*Sce*I (lanes 2-21 and 23-42, respectively). The molecular weight marker is Midrange I (New England Biolabs).

To determine if intact BAC inserts are easily transferred into the BIBAC vector, we tested two BAC clones with inserts of 100 kb and 50 kb, which were picked from a maize Mo17 BAC library that was constructed using the pIndigoBAC536-S vector. The I-*Sce*I digestion products of the two BAC clones were directly ligated, without isolation of the inserts, into the BIBAC-S vector that was prepared from the pHZAUBIBAC1 with I-*Sce*I. The I-*Sce*I recognition site is non-palindromic, and pIndigoBAC536-S and BIBAC-S vectors that are prepared with I-*Sce*I contain the same non-complementary ATAA ends and cannot be ligated or concatenated to themselves or to each other (Figure 1). The pIndigoBAC536-S vector contains a chloramphenicol-resistance gene, and BIBAC-S contains a kanamycin-resistance gene. When transformants are plated on kanamycin- and X-gal-containing selection medium, only those colonies harboring the BIBAC-S vector ligated with the a BAC insert will grow, whereas colonies harboring any original BAC clones that are not completely digested or are reconstituted will not grow. X-gal is added to the selection medium to distinguish the transformants of pHZAUBIBAC1 (blue) that is possibly trapped during the preparation of BIBAC-S from pHZAUBIBAC1. We analyzed 20 white colonies for each ligation. Figure 5 shows that all except one of the BIBAC colonies contain intact BAC inserts. The one empty BIBAC vector colony is a pHZAUBIBAC1 colony carelessly picked during analysis. The ~3.2 kb stuffer fragment (pGEM-4Z plus the *lac*Z gene) of the pHZAUBIBAC1 was run out of the gel. Intact inserts from the BIBAC libraries that are constructed with our BIBAC-S vector can be transferred to the BAC vector pIndigoBAC536-S using the same approach.

**Figure 6:**
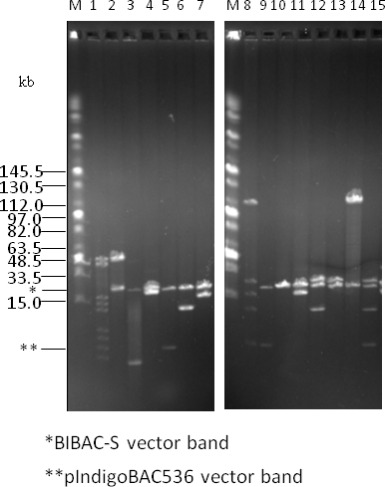
**Transfer of BAC insert fragments into the BIBAC-S vector using *Not*I**. Two BAC clones with insert sizes of ~162 kb (lane 1) and ~165 kb (lane 8) were randomly selected from a rice variety Minghui 63 BAC library that was constructed using the pIndigoBAC536 vector. DNA samples were digested with *Not*I and were ligated to the dephosphorylated BIBAC-S vector that was prepared with *Not*I from the composite BIBAC vector pHZAUBIBAC1. The ligation products were used to transform *E. coli *DH10B competent cells. DNA samples from thirty randomly selected colonies for each ligation were analyzed using *Not*I, and the clones that contain BAC insert fragment(s) (with the exception of lane 10) are shown (lanes 2-7 and 9-15, respectively). The molecular weight marker is Midrange I (New England Biolabs).

To facilitate the transfer of inserts from BAC clones constructed using previous vectors, such as pIndigoBAC536, into the BIBAC-S vector; we retained the two *Not*I sites in the BIBAC-S vector (Figure 1) and tested the feasibility of this approach using two BAC clones. The two BAC clones were randomly picked from the rice variety Minghui 63 BAC library that was constructed using the pIndigoBAC536 vector prepared from pCUGIBAC1 [37,41]. The first clone (Figure 6A, lane 1) produced 8 insert bands following *Not*I digestion with a cumulative insert size of ~162 kb. The second clone (Figure 6B, lane 8) produced 4 insert bands following *Not*I digestion with a cumulative insert size of ~165 kb. Both clones produced a 6.9 kb vector band. The *Not*I digestion products of each BAC clone, without isolation of the insert fragments, were directly ligated to the BIBAC-S vector that was prepared from pHZAUBIBAC1 by *Not*I digestion and dephosphorylation. The ligation products should produce a mixture of self-ligated pIndigoBAC536 vector, pIndigoBAC536 vector ligated with different insert fragment(s), self-ligated incompletely dephosphorylated BIBAC-S vector (empty BIBAC-S vector), BIBAC-S vector ligated with different insert fragment(s) (BIBAC-S clones) and different kinds of concatenates. On kanamycin and X-gal selection medium, only transformants harboring the empty BIBAC-S vector plasmids and the BIBAC-S clones can grow. Because the empty BIBAC-S vector plasmids are a result of self-ligation of the *Not*I backbone fragments that do not contain the *lac*Z gene (Figure 1), their colonies are white in color on X-gal medium, the same as those of the BIBAC-S clones. We analyzed DNA from 30 white colonies for each ligation. For the first ligation, 5 BIBAC-S clones containing different single *Not*I BAC insert fragments (Figure 6A, lanes 2, 4-7) and one containing the BAC vector pIndigoBAC536 (Figure 6A, lane 3) were observed. For the second ligation, 3 BIBAC-S clones containing different single *Not*I BAC insert fragments (Figure 6B, lanes 11, 13-14), one containing two fragments (Figure 6B, lane 12), one containing two fragments and the BAC vector pIndigoBAC536 (Figure 6B, lane 15), and one containing only the BAC vector pIndigoBAC536 (Figure 6B, lane 9) were observed. The data indicate that BIBAC-S clones that contain individual *Not*I fragments can be obtained from a ligation of a mixture, but deep screening and efficient dephosphorylation of the BIBAC-S vector to reduce the number of empty BIBAC-S vector transformants is required.

### Stability of BIBAC clones in E. coli and Agrobacterium

**Figure 7:**
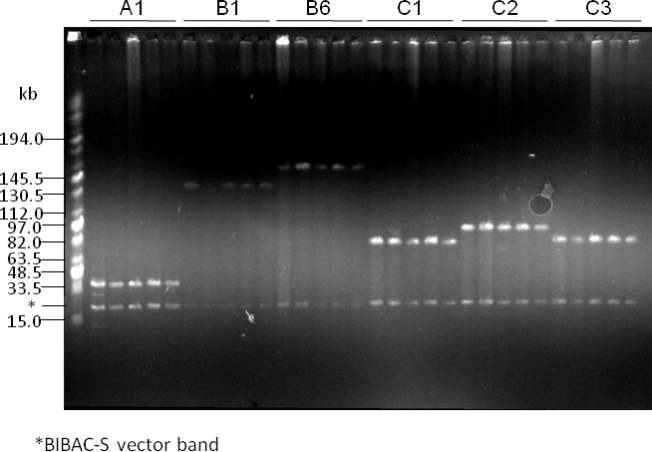
**Stability test for maize B73 BIBAC clones in *E. coli***. BIBAC clones were randomly selected from the maize B73 BIBAC library that was constructed using the BIBAC-S vector. DNA samples were prepared from cultures grown for 16 h, 24 h, 48 h, 72 h and 96 h at 37°C (sub-cultured every 24 h), and were digested with I-*Sce*I and separated on CHEF gels (the samples were loaded in order for each clone). The six BIBAC clones shown (from left to right) are A1, B1, B6, C1, C2 and C3, which contain inserts of about 40 kb, 140 kb, 160 kb, 80 kb, 100 kb and 80 kb, respectively. The molecular weight marker is Midrange I (New England Biolabs).

To test the stability of BIBAC clones constructed using the BIBAC-S vector in *E. coli*, we analyzed the DNA of 9 randomly picked BIBAC clones from the maize B73 BIBAC library. DNA samples were prepared from cells cultured at 37°C for 16 h, 24 h, 48 h, 72 h and 96 h (sub-cultured every 24 h), respectively, were digested with I-*Sce*I and were separated on CHEF gels. Figure 7 shows the results of 6 clones. The restriction patterns of all the clones, including two clones that contained large inserts of 140 kb and 160 kb, did not change after 96 h of culture, which corresponds to more than 200 generations. These results indicate that the BIBAC clones, large or small, are stable in *E. coli*.

**Figure 8:**
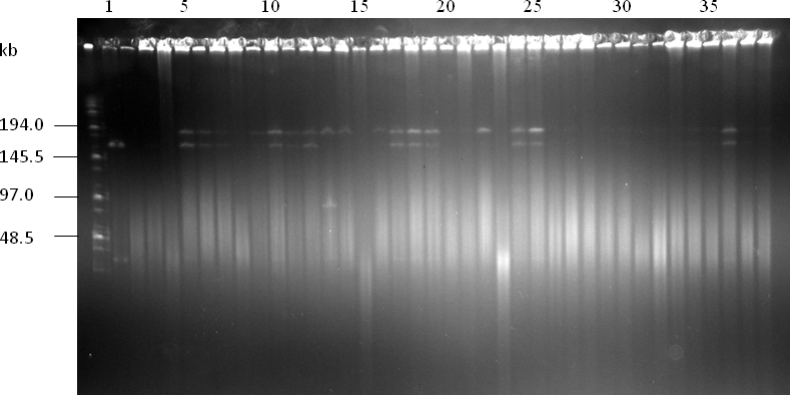
**Stability test for maize B73 BIBAC clones in *Agrobacterium*: direct method**. DNA samples from the maize B73 BIBAC clone B6 that contains an insert of 160 kb (lane 1; see also Figure 7) was transferred into *Agrobacterium *EHA105, and randomly selected colonies (lanes 2 to 38) were cultured for 48 h at 28°C. Plasmids were isolated from each culture, were digested with I-*Sce*I and were separated on CHEF gels.

We used two methods to test the stability of BIBAC clones in *Agrobacterium*. In the first method, DNA samples from 9 maize B73 BIBAC clones (4 of which were the same clones as above) were transferred into *Agrobacterium *EHA105, and various numbers of single colonies for each clone were cultured separately for 48 h and 96 h (sub-cultured after 48 h) at 28°C. Plasmids were isolated from each culture, were digested with I-*Sce*I and were separated on CHEF gels. All clones that were tested were stable after culturing for 48 h and 96 h. Figure 8 shows the results of the EHA105 colonies of the BIBAC clone B6 that was cultured for 48 h. This clone contains a 160-kb insert (See also Figure 7). A band of approximately 190 kb from the Ti plasmid was co-isolated and used as an indicator of successful plasmid isolation. From 37 single-colony cultures of the B6 clone, 9 failed to produce plasmid DNA. Of the 28 samples that contained the 190-kb control band, 24 samples also contained the 160-kb BIBAC insert band (85.7%).

**Figure 9:**
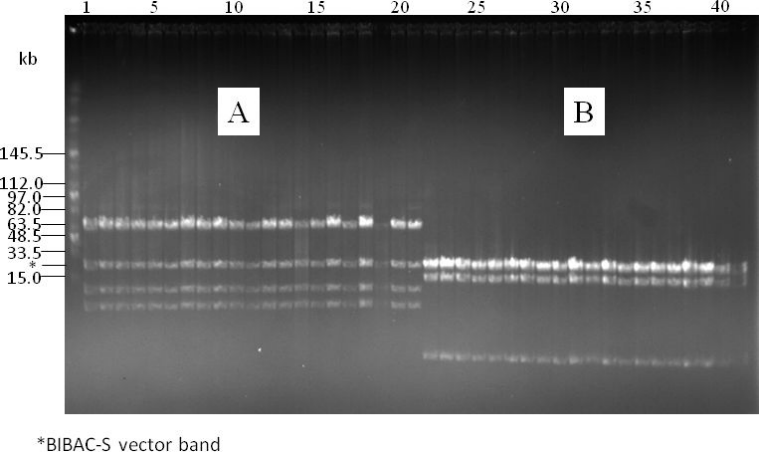
**Stability test for maize B73 BIBAC clones in *Agrobacterium*: indirect method**. DNA samples from the maize B73 BIBAC clones A and B that contain inserts of approximately 95 kb and 20 kb (lanes 1 and 22), respectively, were transferred into *Agrobacterium *EHA105 and were cultured for 96 hours at 28°C. DNA prepared from the *Agrobacterium *cultures was transferred back into *E. coli*, and DNA samples from randomly selected single colonies (lanes 2-21 and 23-41, respectively) were analyzed using *Not*I and were separated on CHEF gels. The molecular weight marker is Midrange I (New England Biolabs).

Because it is difficult to recover enough plasmid DNA from *Agrobacterium *cultures for DNA analysis, in the second method (an indirect method that has been used in other laboratories [22,31,48]), we cultured random *Agrobacterium *colonies for different times, re-transformed the plasmids isolated from these *Agrobacterium *cultures back into *E. coli *and isolated the plasmids from *E. coli *cultures for analysis. We independently analyzed 10 maize BIBAC clones (2 of which were used in the first method) with this method and determined that all of the clones had the expected BIBAC inserts after 96 h (4 days) of growth in *Agrobacterium*. Figure 9 shows the results of two maize BIBAC clones digested with *Not*I. Taken together, our analysis indicates that the new BIBAC vector BIBAC-S and its maize clones, even those containing inserts as large as 140 kb and 160 kb, are stable in *Agrobacterium*.

## Discussion

We constructed a pair of new BAC and BIBAC vectors that can facilitate the construction of large DNA fragment libraries and the release and exchange of intact large DNA inserts between the two vectors.

Previously available BAC and BIBAC vectors usually use *Not*I digestion for insert sizing and release. For large DNA-insert BAC and BIBAC clones from high GC content organisms or monocotyledonous plant genomes, digestion with *Not*I cuts each insert into several to many fragments, making insert sizing difficult and the release of intact inserts almost impossible. Although the available BAC and BIBAC vectors also contain lambda *cosN *and P1 *loxP *sites [1,47] that may not be present in the cloned genomic DNA inserts, these sites are located on the same side of the cloning site and cannot be used to release the inserts. Even though these sites could be used to linearize BAC and BIBAC plasmids, insert sizing that depends on linearizing is not reliable because without the presence of a second band as reference, e.g., the vector band, it is impossible to determine if the plasmids have been linearized successfully. On a CHEF gel, a circular plasmid migrates more slowly than its linear form (our experience) and can lead to the overestimation of plasmid size. Hurwitz et al. [46] estimated BAC insert sizes of large contigs of three genomes closely related to rice by mechanically semi-linearizing plasmids to investigate structural variations between the rice and its closest relatives. In this case, each sample produced two bands (circular and linear forms) on CHEF gels and the lower band (linear form) was used for size determination. However, careful optimization of the method is critical, and repeated experiments may be frequently required to generate two bands in each sample.

We created two I-*Sce*I sites that flank the cloning site in both BAC and BIBAC vectors to release and exchange intact large DNA inserts. When the I-*Sce*I recognition sequence was used to search the genome sequence database, no any sites were found in Arabidopsis and the rice Nipponbare genomes, and only two sites were found in the maize B73 genome. Every BAC and BIBAC clone, irrespective of how large an insert it contains, will release only one insert band (with the exception of the few sites in maize or, possibly, in other genomes), making insert sizing simple. More importantly, intact inserts can be exchanged easily and efficiently between BAC and BIBAC vectors because these vectors use different antibiotic selection markers and both produce the same non-complementary 3' protruding ATAA ends by I-*Sce*I that suppress self- and inter-ligations (Figure 1). Purification of the insert from one vector, which is difficult for large DNA fragments, is not required before ligation into the other vector. Re-cloning of BAC inserts into the BIBAC vector is usually required for gene function complementation. Our recently constructed BAC libraries were constructed using our new BAC vector http://GResource.hzau.edu.cn and allow the convenient transfer of inserts. Our BIBAC vector, which has retained the two *Not*I sites flanking the cloning site, can be used to sub-clone inserts from BAC clones that were constructed using other BAC vectors (Figure 1). However, because the backbone BIBAC vector prepared with *Not*I can self-ligate and can host multiple small fragments but does not include the *laz*Z gene for recombinant clone selection, both the efficient dephosphorylation of the vector (to prevent self-ligation) and deep screening for clones of interest are required.

BAC libraries, especially those for very important basic and public applications such as physical mapping and BAC by BAC genome sequencing, are usually arrayed and stored in single clones in 384-well plates. The arrayed clones have a possibility to be contaminated by other BAC clones during picking, replicating and repeatedly using. If a well contains two or more clones by contamination, the sample from the well will fail to produce BAC end sequences and will produce fingerprints that cause misassemblies of contigs. Therefore, the rate of contaminated wells should be an important parameter for the quality of BAC libraries. However, all the previously published BAC libraries except for the barley BAC libraries published recently [7], to our knowledge, were not evaluated for this parameter due to the technical difficulty. Schulte et al. [7] reported 5 barley BAC libraries that were constructed with genomic DNA fragments prepared using different restriction enzymes or mechanical shearing. These authors produced fingerprint files for about 10,000 wells of each library and compared the fingerprint files between neighboring wells of the same plate or between the identical wells of the neighboring plates. The well was considered to be potentially contaminated if its fingerprint profile contains > 50% of fragments identical to the other fingerprint profile. From one BAC library that was constructed earlier, the potential neighbor and plate-wide contamination were estimated to be 2.73% and 7.28%, respectively. From the newly constructed four BAC libraries, the potential neighbor and plate-wide contamination were estimated to be from 1.01% to 2.09%, and from 1.44% to 5.76%, respectively. However, this method may not be practical to most BAC libraries. Fingerprinting is a costly work and so before that the quality of the BAC library should be already determined. Also, this method determines the potential contamination of a well depending on not only the fingerprint profile of this well but also the fingerprint profiles of the contamination source wells. With this method, the wells that are contaminated by non-arrayed clones or by the arrayed clones that do not have successful fingerprint profiles cannot be determined. Our new vectors can solve the problem. For the BAC and BIBAC libraries constructed with our new vectors, rates of wells that contain two or more clones can be estimated during quality evaluation of the library with I-*Sce*I. If the DNA sample of a well produces two or more insert fragments by I-*Sce*I digestion, the well can be considered to contain two or more clones. If necessarily, the inserts of the single colonies streaked out from the flagged well can be re-analyzed with I-*Sce*I for validation.

BAC libraries that are constructed with genomic DNA fragments prepared using restriction enzymes suffer from cloning bias due to the uneven distribution of the restriction sites [7,24,49]. The genomic regions that contain few or none restriction sites for the enzymes that are used in BAC library construction are underrepresented or missed in the BAC libraries. To reduce cloning bias, complementary BAC libraries with genomic DNA fragments prepared using different restriction enzymes or mechanical shearing are usually required. Osoegawa et al. [49] established a system to construct BAC libraries with randomly sheared large genomic DNA fragments. These researchers developed a BAC vector (pTARBAC6) that contains two *Bst*XI recognition sites (CCATTGTGTTGG) in an inverted orientation at the positions flanking a stuffer fragment. After digestion by *Bst*XI, the vector produces two 3' protruding TGTG ends that are not complementary to each other. During BAC library construction, adaptors containing 3' protruding CACA ends that are not complementary to each other but are complementary to the vector ends are added to the randomly sheared and polished large genomic DNA fragments. With this system, several *Drosophila *BAC libraries [49] and a barley BAC library [7] were constructed. The former has been used to close physical gaps and clone telomeric regions. Both our new BAC and BIBAC vectors contain 3' protruding non-complementary ATAA ends when prepared with I-*Sce*I and could be used to construct BAC and BIBAC libraries with randomly sheared large genomic DNA fragments using the same approach as above except for changing the adaptor to that containing 3' protruding TTAT end.

Both of the new low-copy BAC and BIBAC vectors, pIndigoBAC536-S and BIBAC-S (1-2 copies/cell), were made into high-copy vectors by constructing composite plasmids using the high-copy vector pGEM-4Z following a previously described strategy [37]. The two high-copy composite vectors, pHZAUBAC1 and pHZAUBIBAC1, facilitate the efficient preparation of the normally low-copy BAC and BIBAC vectors pIndigoBAC536-S and BIBAC-S, respectively, and contamination of the high-copy plasmids in the BAC/BIBAC libraries will not occur, due to the special features incorporated into the composite vectors [37].

Previously available BIBAC and TAC vectors [22,47] use the *Sac*B gene to select recombinant clones. In our strategy, the *lac*Z gene is used in both of the pIndigoBAC536-S and BIBAC-S vectors in order to reconstitute the new *lac*Z genes with the *lac*Z gene of the pGEM-4Z vector in the high-copy composite vectors. In our experience, the *lac*Z gene is a useful marker because it produces a visible color on selection medium. The construction of large DNA fragment libraries is a high-throughput endeavor and negligence, however trivial, at any step can affect the final library quality. Using the *lac*Z gene selection system, leaky background colonies can be distinguished and eliminated. The ratio of blue to white colonies in pilot experiments can be used to evaluate the quality and efficiency of the vector preparation steps, e.g., restriction enzyme digestion, dephosphorylation and gel separation. Conversely, the presence of background blue colonies is sometimes an indicator of correct medium preparation. Indeed, Chang YL et al. [30] reported that BIBAC libraries constructed with the *Sac*B selection system usually contained higher numbers of empty vector clones than did the BAC libraries constructed with the *lac*Z selection system. The Arabidopsis BIBAC library [30] and the tomato BIBAC library [28], constructed using the original BIBAC2 vector with the *Sac*B selection system, contained 17.6% and 13% of empty-vector clones, respectively, whereas BAC libraries constructed using the BAC vector with the *lac*Z selection system usually contained less than 5% empty-vector clones. The maize and sorghum BIBAC libraries that were constructed using our new BIBAC vector BIBAC-S with the *lac*Z selection system had a low percentage of empty vector clones (less than 2%; data not shown).

Although most BAC and BIBAC clones were reported to be stable in *E. coli *and most BIBAC clones were stable in *Agrobacterium *[17,21,22,32,37], Song et al. [48,50] reported that BAC clones containing tandem repeat DNA sequences were not stable in *E. coli *and that BIBAC and TAC clones containing potato genomic DNA fragments larger than 100 kb were not stable in *Agrobacterium*. Liu YG et al. [22,31] reported that one out of 35 TAC clones containing Arabidopsis DNA fragments of < 100 kb was not stable, while 6 out of 16 TAC clones containing wheat DNA fragments of ~150 kb were not stable. We tested the stability of maize B73 BIBAC clones with insert sizes ranging from 40 kb to 160 kb in *E. coli *and *Agrobacterium*. The BIBAC clones were stable in *E. coli *and were considerably stable in *Agrobacterium *after at least 96 h (4 days) of growth. When DNA plasmids purified from *Agrobacterium *were directly analyzed, some samples of the BIBAC clones contained shorter or none inserts (e.g., Figure 8), a sign of instability. However, at least some of these samples may be a result of poor preparation of the low-copy large BIBAC DNA from *Agrobacterium*. Obtaining enough low-copy large BIBAC DNA from *Agrobacterium *was difficult, especially when handling large numbers of parallel samples. When the independent indirect method was used, all of the DNA samples from the BIBAC clones contained the expected inserts. Stable maintenance of BIBAC/TAC clones in *Agrobacterium *is a prerequisite for *Agrobacterium*-mediated transformation. Factors affecting the stability of BIBAC/TAC clones in *Agrobacterium *are not known although large insert size and highly repetitive sequences in the clones are suspected to be the most probable cause of instability [31,48]. Our new BIBAC vector BIBAC-S contains two identical 26-bp I-*Sce*I-*Not*I sequences flanking the cloning sites. The maize genome is known to contain highly repetitive sequences [11]. However, large fragments of maize DNA cloned into the BIBAC-S vector are stable, indicating that the large insert size and highly repetitive sequences may not necessarily affect the stability of BIBAC/TAC clones in *Agrobacterium*. In fact, the 160-kb B6 clone and many others were completely transferred into rice via *Agrobacterium *(Manuscript in preparation).

## Conclusions

We have developed a pair of new BAC and BIBAC vectors and made the two low-copy vectors into the high-copy composite vectors. The two new vectors and their respective high-copy composite vectors can largely facilitate the construction and characterization of BAC and BIBAC libraries. The transfer of complete large genomic DNA inserts from one vector to the other is made straightforward.

## Materials and methods

### Construction of the new BAC vector pIndigoBAC536-S

PCR was performed to amplify the *Not*I fragment (containing the *lac*Z gene and cloning sites) of the pIndigoBAC536 plasmid using the forward primer P1, 5'-AAGGTCGACtagggataacagggtaatCGTCAGCGGGTGTTGGCGG-3' and the reverse primer P2, 5'-CCTGTCGACtagggataacagggtaatAGGGGTTCGCGTTGGCCGAT-3'. Both primers contain a *Sal*I site (underlined) and an I-*Sce*I site (lower case) at the 5' ends. The pIndigoBAC536 plasmid that was used as the PCR template was originally provided by Dr. M. Simon of Caltech, CA, USA, and was recovered from pCUGIBAC1 [37]. The PCR product was cloned into the pGEM-T Easy vector (Promega), and its sequence was confirmed. The insert was recovered by *Sal*I digestion. Because the *lac*Z gene contains an internal *Sal*I site, the insert was digested into two *Sal*I fragments of 371 bp and 282 bp. The *Sal*I-digested backbone BAC vector fragment of 6384 bp that was recovered from the pIndigoBac536 plasmid by *Sal*I digestion was dephosphorylated with CIAP phosphatase and was ligated with the two *lacZ Sal*I fragments above. The ligation products were used to transform DH10B-competent cells. Transformants were selected on LB medium containing chloramphenicol (12.5 μg/mL), X-gal (5-bromo-4-chloro-3-indolyl-β-D-galactopyranoside, 80 μg/mL) and IPTG (Isopropyl-β-D-thiogalactopyranoside, 100 μg/mL). Blue colonies should contain one of two different ligation products, each containing a complete *lac*Z gene with opposite orientations relative to the vector backbone. The blue colonies were further analyzed, and the clone with the *lac*Z gene in an orientation relative to the vector backbone that was similar to the original pIndigoBAC536 was selected. Two *Nde*I sites, one located in the *lac*Z gene and the other in the vector backbone, were used to distinguish the two ligation products. Digestion of plasmids from the required ligation product with *Nde*I resulted in 4280 bp and 2757 bp fragments, whereas plasmids from the non-required ligation product yielded 3820 bp and 3217 bp fragments. The restriction sites *Sal*I, I-*Sce*I, *Hin*dIII, *Bam*HI and *Eco*RI in the new vector were validated by digestion.

### Construction of the new BIBAC vector BIBAC-S

The same *Not*I fragment of the pIndigoBAC536 plasmid described above was amplified by PCR using the forward primer P3, 5'-AAGGAAAAAAGCGGCCGCtagggataacagggtaatCGTCAGCGGGTGTTGGCGG-3' and the reverse primer P4, 5'-AAGGAAAAAAGCGGCCGCAtagggataacagggtaatAGGGGTTCGCGTTGGCCGAT -3'. Both primers contain a *Not*I site (underlined) and an I-*Sce*I site (lower case) at the 5' ends. The PCR product was cloned into the pGEM-T Easy vector (Promega) and its sequence was confirmed. The insert was recovered by *Not*I digestion and was ligated to the dephosphorylated *Not*I-digested backbone BIBAC vector fragment that was prepared from BIBAC2 ([47]; obtained from the Cornell Center for Technology Enterprise & Commercialization). The ligation products were transformed into DH10B-competent cells. Transformants were selected on LB medium containing kanamycin (20 μg/mL), X-gal (80 μg/mL) and IPTG (100 μg/mL). The blue colonies, which should contain the *lac*Z gene (PCR product), were further analyzed. The clone that contained the *lac*Z gene in an orientation to the vector backbone as shown in Figure 1 was selected. The restriction sites I-*Sce*I, *Not*I and *Bam*HI of the new vector were validated by digestion.

### Construction of the high-copy composite BAC vector pHZAUBAC1 and BIBAC vector pHZAUBIBAC1

The high-copy composite vectors were constructed following a previously described approach [37]. The high-copy composite BAC vector pHZAUBAC1 was constructed by ligating the low-copy BAC vector pIndigoBAC536-S to the high-copy pGEM-4Z at the *Hin*dIII site, and was selected on LB medium containing chloramphenicol (12.5 μg/mL), ampicillin (50 μg/mL), X-gal (80 μg/mL) and IPTG (100 μg/mL). The high-copy composite BIBAC vector pHZAUBIBAC1 was constructed by ligating the low-copy BIBAC-S to the high-copy pGEM-4Z at the *Bam*HI site, and was selected on LB medium containing kanamycin (20 μg/mL), ampicillin (50 μg/mL), X-gal (80 μg/mL) and IPTG (100 μg/mL). All resulting colonies were to be blue.

### Re-cloning of intact BAC inserts into the BIBAC-S vector

Plasmid DNA from maize Mo17 BAC clones that were constructed using the new BAC vector pIndigoBAC536-S was extracted using the Qiagen plasmid preparation kit (Qiagen) and was digested with I-*Sce*I for 5 hours at 37°C. The samples were heated at 70°C for 10 min to inactivate the enzyme and were extracted once with chloroform. The BAC digestion products were precipitated with ethanol and resuspended in ddH2O. The I-*Sce*I-digested backbone BIBAC-S vector (23.2 kb) was prepared by digesting the high-copy composite BIBAC vector pHZAUBIBAC1 with I-*Sce*I followed by separation of the digestion products on a 1% agarose gel and electroelution of the 23.2 kb DNA band from the gel. The I-*Sce*I-digested backbone BIBAC-S vector was ligated to the I-*Sce*I BAC digestion products at 16°C overnight. The ligation products were used to transform DH10B-competent cells. The transformants were selected on LB containing kanamycin (20 μg/mL), X-gal (80 μg/mL) and IPTG (100 μg/mL). DNA plasmids prepared from the resulting white colonies were digested with I-*Sce*I and were analyzed using pulse-field gel electrophoresis.

### Re-cloning of NotI fragments of BAC inserts into the BIBAC-S vector

The *Not*I BAC digestion products were prepared following the same procedures as described above for the I-*Sce*I BAC digestion products. Rice MH63 BAC clones constructed using the BAC vector pIndigoBAC536 prepared from pCUGIBAC1 [41] were chosen for this experiment. The *Not*I-digested backbone BIBAC-S vector (23.2 kb) was prepared by digesting the high-copy composite BIBAC vector pHZAUBIBAC1 with *Not*I, dephosphorylating the digestion products with CIAP, separating the digestion products on a 1% agarose gel and electroeluting the 23.2 kb DNA band from the gel. The dephosphorylated *Not*I-digested backbone BIBAC-S vector was ligated to the *Not*I BAC digestion products at 16°C overnight. Subsequent procedures were carried out as described above. DNA plasmids from the resulting white colonies were digested with *Not*I and were analyzed using pulse-field gel electrophoresis.

### Stability tests of BIBAC clones in E. coli and Agrobacterium

To test the stability of BIBAC clones in *E. coli*, the clones were cultured in LB medium containing 20 μg/mL kanamycin at 37°C with shaking at 250 rpm and were sub-cultured every 24 hours. Plasmid DNA was extracted and was analyzed using pulse-field gel electrophoresis. To test the stability of BIBAC clones in *Agrobacterium*, BIBAC DNA was transferred into the *Agrobacterium *strain EHA105 by electroporation. EHA105 colonies were chosen at random, were cultured in LB medium containing 20 μg/mL kanamycin at 28°C with shaking at 250 rpm and were sub-cultured every 48 hours. In the direct-test experiment, DNA plasmids were extracted from EHA105 cultures and were analyzed using pulse-field gel electrophoresis. In the indirect-test experiment, DNA plasmids were extracted from EHA105 cultures and were transferred back into *E. coli *DH10B cells. Following propagation in *E. coli*, plasmid DNA was re-extracted from the *E. coli *cultures and was analyzed using pulsed-field gel electrophoresis.

### Pulsed-field gel electrophoresis

BAC or BIBAC DNA plasmids were prepared from *E. coli *or *Agrobacterium *cultures, were digested with I-*Sce*I or *Not*I as indicated and were separated on 1% agarose CHEF (CHEF-DRIII apparatus, Bio-Rad) gels at 6 V/cm and 14°C in 0.5 × TBE buffer with a linear ramp time from 5 to 15 s for 16 h.

## List of abbreviations

BAC: bacterial artificial chromosome; BIBAC: binary BAC; TAC: transformation-competent artificial chromosome; YAC: Yeast artificial chromosome IPTG: Isopropyl-β-D-thiogalactoside; X-gal: 5-bromo-4-chloro-3-indolyl-β-D-galactopyranoside.

## Competing interests

The authors declare that they have no competing interests.

## Authors' contributions

ML designed the experiments, analyzed the data and wrote the manuscript. XS constructed the vectors and tested their utilities. HZ investigated the stabilities of BIBAC clones in *E. coli *and *Agrobacterium*. YX attended the initial experiments. All authors read and approved the final manuscript.
